# Association between Dental Caries and Down Syndrome: A Systematic Review and Meta-Analysis

**DOI:** 10.1371/journal.pone.0127484

**Published:** 2015-06-18

**Authors:** Tahyna Duda Deps, Gabriela Lopes Angelo, Carolina Castro Martins, Saul Martins Paiva, Isabela Almeida Pordeus, Ana Cristina Borges-Oliveira

**Affiliations:** 1 Department of Pediatric Dentistry and Orthodontics, Faculty of Dentistry, Universidade Federal de Minas Gerais, Belo Horizonte, Minas Gerais, Brazil; 2 Department of Social and Preventive Dentistry, Faculty of Dentistry, Universidade Federal de Minas Gerais, Belo Horizonte, Minas Gerais, Brazil; LSU Health Sciences Center School of Dentistry, UNITED STATES

## Abstract

Scientific evidence of susceptibility to dental caries in the population with Down Syndrome (DS) is limited and conflicting, making it difficult to establish firm conclusions. The aim of this systematic review and meta-analysis was to obtain scientific evidence of the possible association between dental caries and individuals with DS, compared to individuals without DS (control). An electronic search of five databases was performed, with no language or publication date restrictions. The studies were selected by two independent reviewers (Kappa = 0.83). The systematic review included 13 studies, while eight studies were included in the meta-analysis. The studies are presumably all at risk of bias given their observational character. Two of these evaluated the presence or absence of caries in permanent and deciduous teeth, and six evaluated the mean DMFT index in permanent teeth. Combined odds ratios (OR), standard difference, standard error and a 95% confidence interval (CI) were obtained. The vast majority of the studies found that individuals from control groups had more carious lesions or caries experience than those with DS. The results were statistically significant in seven studies (p<0.05). Meta-analysis of two studies revealed that individuals with DS had a lower dental caries than those in the control group (OR = 0.36; 95% CI = 0.22–0.57). In six studies, individuals with DS had a significantly lower mean DMFT index than individuals from the control group (Sd = -0.18; SE = 0.09; 95% CI = -0.35–-0.02). The quality of the studies varied and in general had a high risk of bias. Scientific evidence suggests that individuals with DS have fewer dental caries than individuals without DS.

## Introduction

Down syndrome (DS) is one of the most common genetic abnormalities, and has a highly variable prognosis. Individuals with DS have specific orofacial characteristics associated with the syndrome. The most common oral disorders include periodontal disease, malocclusion, mouth breathing, macroglossia, delayed teeth eruption, missing and malformed teeth, microdontia, diastema and bruxism [1–6].

Scientific evidence of susceptibility to dental caries in the population with DS is limited and conflicting, making it difficult to establish firm conclusions [7–9]. The vast majority of published studies report a lower prevalence and experience of caries in this group of individuals than in groups not affected by DS and groups with other disabilities [6,10–17].

A smaller number of studies, however, have highlighted an equivalent or higher prevalence of caries in individuals with DS [18–21]. According to these authors, some local factors determinant of caries (difficulty of access to dental care, poor dietary habits, use of drugs for severe infections of the upper airways, reduced manual dexterity, poor oral hygiene, parental neglect) override "protective factors" (such as the buffer capacity of saliva, bruxism, diastema, agenesis and microdontia).

A systematic review conducted with 27 studies has reported that patients with intellectual disabilities presented poorer oral hygiene and more prevalence of periodontal disease than control patients. Also, these patients had caries rates equal or lower than the general population [22]. However, several disabilities were pooled together to base the conclusions: mental retardation, developmental disabilities, intellectual disabilities, DS, handicapping and autism. Considering the conflict of studies involving the prevalence of caries in the population with DS, the absence of a systematic review on this issue justifies the present study.

In view of the various dentofacial alterations present in individuals with DS, defining the real situation of the caries disease is important for identifying the priorities that should be given to dental care for this segment of the population. Therefore, the aim of the present study was to perform a systematic review and meta-analysis of the literature, to seek scientific evidence of a possible association between dental caries in individuals with DS, compared to individuals without DS (control).

## Materials and Methods

The clinical question [PICO question: Patient/Problem, Intervention, Comparison, Outcome (evidence-based medicine)] was: individuals (Patients); DS (Exposure to risk factor/Intervention); healthy condition (Comparison); dental caries (Outcome).

### Search Strategy

The present study included observational epidemiological studies (cross-sectional, case-control, cohort) that assessed the prevalence, incidence or experience of dental caries in individuals (no age limit) with DS compared with a control group of individuals without DS. The studies are presumably all at risk of bias given their observational character.

An electronic search of five electronic databases by three researchers (TDD, GLA and CCM) was performed in March 2013 and updated in September 2014. The databases were: Medline via PubMed (http://www.ncbi.nlm.nih.gov/pubmed), Web of Science (www.isiknowledge.com), Cochrane Library (http://www.thecochranelibrary.com), Brazilian Library of Dentistry (BBO) and the Latin American and Caribbean System of Health Sciences, via Bireme (www.bireme.br). There was no restriction on language or date of publication. A manual search of the lists of references of articles selected for reading was also conducted.

For Pubmed, Web of Science and Cochrane the following search strategy was used: ((caries OR Dental Caries [Mesh] OR dental decay OR DMF index [Mesh] OR decayed teeth) AND (Down Syndrome [Mesh] OR trisomy 21 OR mongolism OR trisomy 21, meiotic nondisjunction OR trisomy 21, mitotic nondisjunction OR partial trisomy 21 OR down syndrome*)). For BBO and Lilacs the keyword combinations “down syndrome” and “dental caries” were used.

The studies were imported into Reference Manager (Reference Manager, Thomson Reuters, version 12.0.3). A total of 217 studies were obtained from the electronic databases, of which 153 were read to check their eligibility for inclusion and exclusion criteria, after the removal of duplicates (64) (S1 Fig).

Exclusion criteria included studies reporting on the prevalence, incidence or experience of dental caries in individuals with DS without a control group, case report or report of series of cases, prevalence of caries related to other syndromes, literature review, study without statistical analysis, outcome different from dental caries. Reviews were fully read and analyzed in order to try to identify studies not retrieved by electronic search (manual search). A manual search was also conducted on the reference list of included studies. However, literature reviews were excluded from the systematic review. S1 Table presents the excluded studies from the full text analysis.

The studies were selected by two independent researchers calibrated for inclusion and exclusion criteria (TDD and GLA). An initial calibration was performed with 20% of the studies, which resulted in a level of agreement that was considered to be very good (Kappa: 0.83). Following this step, the researchers continued to read the rest of the studies independently [23] (S1 Fig).

When it was not possible to obtain studies to read or when additional data was required, the authors were contacted by email. Abstracts of studies published at scientific events were also found. When these were of interest, the authors were contacted in an attempt to obtain the full text. However, only one author [24] (out of seven authors contacted) responded by sending the full text.

### Data Extraction

Data extraction was performed by two independent researchers (TDD and GLA). The following data were extracted: publication language, local setting in which data collection was undertaken, sample size, age, index used for dental caries, statistical analysis and results obtained.

Eight studies were included in the meta-analysis. The articles were sorted into two groups in accordance with the description of the authors. Six studies assessed dental caries in permanent teeth, with the mean DMFT index (Decayed, Missing, and Filled Teeth) being the dependent variable analyzed [13,15,17,20,24,25]. Two studies considered all teeth (deciduous and permanent) and used a categorical dependent variable (presence or absence of dental caries) [6,14,15]. A comparison was made between individuals with DS and control subjects.

### Methodological Quality Assessment

Study quality was assessed using the Newcastle-Ottawa Scale modified for cross-sectional studies [26]. The following criteria were assessed: selection of study groups (confirmation of diagnosis of DS by genetic testing; selection of individuals with DS from referral centers, control patients without disabilities); control for confounding factors (medications, socioeconomic status); and outcome assessment (assessment of dental caries by a previously calibrated examiner; clinical evaluation of tooth decay; same evaluation method for cases and controls; non-response rate). For each criterion completed the study received scores ranging from 0 to 8.

### Statistical Methods and Data Synthesis

The Comprehensive Meta-Analysis software program, version 2, was used for meta-analysis [27]. The heterogeneity of studies was evaluated using the I^2^ test [28]. A sensibility test was conducted when heterogeneity was above 50% [29]. The random effect model was used for meta-analysis in all cases, because when studies are gathered from the published literature, the random effects model is generally a more plausible match [30]. To estimate the variance of true standardized mean differences (τ^2^), DerSimonian and Laird method was used [29]. For categorical data (presence of dental caries vs. absence of dental caries) summary risk measures were defined by odds ratio (OR). For continuous data (mean of DMFT in permanent dentition), standard difference (Std diff) was used. For both forest plots, 95% confidence interval (CI) and p-values were calculated. Publication bias was not assessed as there were not enough studies for inclusion in a funnel plot [30].

## Results

### Studies Characteristics

Twenty-six studies were selected for full-text analysis, with 13 studies included in the systematic review (13 cross-sectional studies featuring a comparison group), and eight in the meta-analysis (S1 Fig).

Table 1 shows the characteristics of the studies. The studies were from India [25], Jordan [17], Portugal [6,14,15], Brazil [24], Turkey [11,31], the USA [32,33], China [13], Nigeria [20] and the Republic of Korea [34]. Although they presented different results, the studies by Areias (2011; 2012) used the same sample group [14,15].

**Table 1:** Study characteristics of 13 cross-sectional studies included in systematic review.

Author (Date)	Country	Local Setting	Sampling Total (cases and controls)	Age of Subjects (in years) or (mean±standard deviation in years)	Measures for Dental Caries (calibration)	Statistics	Results for Dental Caries mean (standard deviation); p-value	Newcastle-Ottawa Quality (total)
Mathias et al., 2011 [24]	Brazil	Cases: 3 reference centers of patients with DS*; Control: Private School	138 (69 cases with DS* and 69 controls)	Cases: (1–7); Control: (NR**)	DMFT^†^ (K^‡^ = 0.89 intra-examiner)	t-test and chi-squared and Fishers	DS*: 2.2 (6.3); Control: 3.4 (8.1) / p = 0.345	7 / 8
Kocht et al., 2010 [33]	USA	Cases: Reference center; Control: NR**	289 (55 cases with DS*, 74 cases with mental disability and 88 controls)	SD cases: (18–56); Mental disability Cases: (22–84); Controls: (18–73)	Missing teeth (K^†^ = 0.93, intra-examinier; K^‡^ = 0.81, inter-examiner)	t- test, chi-squared, Spearmans, correlation analysis, linear regression	SD*: 4.6 (0.52); Control: 1.8 (0.41) / p<0.001	7 /8
Subramaniam et al., 2014 [25]	India	Cases: Special School Control: Department of Pedodontics and Preventive Dentistry	68 (34 cases with SD*, 34 case controls)	SD: (7–12); Control: (7–12)	DMFS^††^ deciduous and permanent—one examinator; (K^‡^ = 0.81, inter-examiner)	Kruskal-Wallis, Mann-Whitney, Spearman’s correlation	dmft for DS*: 2.69 (1.62) for Control: 2.90 (1.60) / p = 0.559 DMFT for DS* = 1.68 (0.69) for Control: 1.84 (1.12) / p = 0.979	7 / 8
Cogulu et al., 2006 [31]	Turkey	Cases: Reference center of Genetics; Control: Public school	124 (60 cases with DS* and 64 controls)	Cases: (7–12); Control:(7–12)	DMFS^††^ deciduous and permanent—one examinator; (K^‡^ = NR**)	t-test, chi-squared tests, Spearman’s rank correlation coefficient and Mann-Whitney tests	DMFT^†^ DS*: Median: 1.00; Minimum: 0—Maximum 4.00 Range: 4.00; p = 0.026 Control: Median: 3.00; Minimum: 1 Maximum: 3.00 Range: 2 / p = 0.026 dfs^††††^ DS*: Mediam: 1.00 Minimum: 0 Maximum: 8.00 Range: 8.00 / p = 0.012 Control: Median: 4.00; Minimum: 0 Maximum: 12.00 Range: 12 / p = 0.012	6 / 8
Al Habashneh et al., 2012 [17]	Jordan	Cases: Reference center of patients with DS*; Control: Public schools	206 (103 cases with DS* and 103 controls)	Cases: (12–16); Control:(12–16)	DMFT^†^ (K^‡^ = 0.89 intraexaminer)	t-test and chi-squared	DS*: 3.32 (3.77); Control*: 4.59 (4.21) / p = 0.023	5 / 8
Cheng et al., 2007 [13]	China	Cases: Reference center of patients with DS*; Control: NR**	130 (65 cases with DS* and 65 controls)	Cases: (26.8 ±6.4); Controls: (26.6 ±6.5)	DFT^†††^—One examinator with calibation but (K^‡^ = NR**)	Mann-Whitney, chi-square and Fisher’s test	DS*: 3.3 (6.2); Control: 4.4 (3.8) / p = 0.001	5 / 8
Macho et al., 2013 [6]	Portugal	Cases: Reference center of patients with DS*; Control: sibling	224 (138 cases with DS* and 86 controls)	Cases: (2–26); Controls: (2–26)	DMFT^†^—One examinator; (K^‡^ = NR**)	t-test, chi-squared and Mann-Whitney test	DMFT^†^ = 0: for SD* (n = 99); for Control (n = 40) DMFT^†^ >1: SD* (n = 39); for Control (n = 46) / p = 0.001	4 / 8
Areias et al., 2012 [15]	Portugal	Cases: National database; Control: Sibling closest in age	90 (45 cases with DS* and 45 controls)	Cases: (12.7 ±4.0); Control: (12.8 ±3.7)	DMFT^†^ primary and permanente (K^‡^ = NR**)	t-test and chi-squared and Fisher’s test	DS*: 1.02 (2.42); Control: 1.84 (3.13) / p = 0.167	4 / 8
Areias et al., 2011 [14]	Portugal	Cases: National database; Control: Siblings closest in age	90 (45 cases with DS* and 45 controls)	Cases: (6–18); Controls: (6–18)	DMFS^††^—One examinator; (K^‡^ = NR**)	t-test, chi-squared or Fisher’s and Mann-Whitney test	DMFT^†^ = 0: for SD* (n = 35); for Control (n = 26) DMFT^†^>1: for SD* (n = 10); for Control (n = 19) / p = 0.042	4 / 8
Yarat et al., 1999 [11]	Turkey	Cases: Reference center of patients with DS*; Control: Not informed	51 (26 cases with DS* and 25 controls)	Cases: (7–22); Control: (6–24)	DMFS^††^ deciduous and permanent; DMFT deciduous and permanent-one examinator; (K^‡^ = NR**)	t-test and correlation analysis	Caries índices were not significantly different between groups (p>0.5)	4 / 8
Orner, 1975 [32]	USA	Cases: 100 children of public institution, 24 taken at home; Control: Unaffected sibs of DS* children	336 (212 cases with DS* and 124 controls)	Cases: (5–20); Control:(5–20)	DMFT^†^—one examinator; (K^‡^ = NR**)	t-test and chi-squared test	Median: DS*: 1.19; Control: 3.86	4 / 8
Oredugba, 2007 [20]	Nigerian	Cases: Reference center of patients with DS*; Control: Nearby schools and some members of staff of those institutions	86 (43 cases with DS* and 43 controls)	Cases: (14.15±7.84); Controls: (14.15±7.84)	DMFT^†^ deciduous and permanent, number of examinators and (K^‡^ = NR**)	Chi-square and Kruskal-Wallis	dmft for DS: 0.67 (2.0); for Control 0.07 (0.3) / p>0.05 DMFT for DS*: 0.23 (0.64); for Control: 0.09 (0.29) / p>0.05	4 / 8
Lee et al., 2004 [34]	Korea	Cases: NR**; Control: NR**	47 (28 cases with DS* and 19 controls)	Cases: (8–17); Controls: (8–17)	DMFS^††^ deciduous and permanent,-one examinator (K^‡^ = NR**)	t- test	dmft for DS*: 6.84 (8.73); for Control: 34.81 (20.38) / p < 0.01-DMFS for DS*: 4.82 (5.64); for Control: 8.35 (6.25) / p<0.05	3 / 8

DS:*Down Syndrome; NR**: Not Reported; K^‡^: Kappa value; DMFT^†^: decayed, missing, filled teeth; DMFS^††^: decayed, missing, filled surface; DFT^†††^ = decayed, filled teeth; dfs^††††^ = decayed, filled surface

Nine studies recruited participants with DS from specialized centers [6,11,13,17,20,24,25,31,32]. Three sought participants through the national database [14,15,33]. One study did not report how the process of selecting of participants was conducted [34]. Five studies recruited control subjects in schools [17,20,24,25,31], while another four studied siblings of individuals with DS [6,14,15,32]. Four studies did not report how the selection of the control group was carried out [11,13,33,34].

No study clarified the sample size calculation used. All reported sample inclusion and exclusion criteria. The sample size ranged from 47 to 336 subjects, with ages ranging from 1 to 84 years.

In 12 studies, the evaluation of caries experience or dental caries was conducted through clinical exam, in accordance with World Health Organization criteria (WHO) criteria (DMFT, DMFS, dmft, dmfs) [6,11,13–15,17,20,24,25,31,32,34]. Only one study evaluated missing teeth [33].

The vast majority of studies found that control subjects had more carious lesions or caries experience than those with DS, with statistically significant results in seven studies (p<0.05) [6,13,14,17,31,32,34]. Five studies reported a difference that was not statistically significant (p>0.05) [11,15,20,24,25], including the study that identified a higher experience of caries in participants with DS (p>0.05) [20] (Table 1). In this study, individuals with DS presented a mean dmft of 0.67 against a mean of 0.07 for control subjects. For permanent dentition, the mean of DMFT was higher for individuals with DS (0.23) than for controls (0.09). However, the Kruskall-Wallis test did not reveal any statistically significant difference (p>0.05) for any dentition (Table 1). Khocht et al. [33] reported that subjects without DS had a higher rate of missing teeth (p<0.001).

### Data Synthesis

In both studies that were included in the meta-analysis and that assessed the presence or absence of dental caries, individuals with DS had a lower prevalence of dental caries than control subjects (OR = 0.36; 95% CI = 0.22–0.57) [6,14] (S2 Fig).

Six studies examined the variable tooth decay in permanent teeth through mean DMFT, and meta-analysis showed a lower mean DMFT among individuals with DS than among control subjects (Sd = -0.18; SE = 0.09; 95%CI = -0.35–-0.02) (S3 Fig) [13,15,17,20,24,25].

### Quality Assessment

Methodological quality ranged from 3 to 7 points (Table 1). The studies analyzed followed a similar methodological approach: A group of individuals with DS was compared to control subjects. Twelve studies used WHO criteria (DMFT, DMFS, dmft, dmfs) to analyze the rate of dental caries [6,11,13–15,17,20,24,25,31,34]. One study used the criterion of missing teeth [33].

The methodological limitations of the studies included a failure to confirm diagnosis for DS through karyotype examination [11,13–15,17,20,25,32,34]. Also, in some cases participants with DS were not selected from referral centers [14,15,32,34]. Some studies did not include Kappa values for calibration of the examiners who carried out the evaluation of dental caries [6,11,13–15,20,31,32,34]. No study claimed to have controlled confounding factors in multiple analysis (gender, age, socioeconomic status and oral hygiene).

In terms of satisfying methodological quality criteria, all studies used established criteria for diagnosis of caries, and the same criteria for the assessment of caries was used for all individuals with DS and controls. Half of the studies (50.0%) controlled the variable medication or excluded from the sample, via a criterion for exclusion, individuals who were taking medication [13–15,22,24,31,33].

## Discussion

### Assessment of Bias in Included Studies

In this systematic review there was no bias due to language and year of publication, as no limits were placed on the search. All of the selected studies were written in the English language and published from between 1975 and 2014. In addition, a manual search was also carried out of the reference lists of the studies included in this review. In terms of geographical location, the studies were from different parts of the world: Latin America, North America, Europe, Asia and Africa, with no location bias.

### Strength of Evidence

The results of meta-analysis showed that individuals with DS have significantly fewer dental caries than individuals without DS. This evidence was strengthened by the low heterogeneity obtained from the I^2^ (0.00%) (S2 Fig), which showed statistical homogeneity between studies. Furthermore, the studies revealed methodological homogeneity [35].

Several factors in the literature are related to the lower prevalence or experience of caries in this section of the population. One of the most commonly reported refers to orofacial characteristics of individuals with DS [1–6,8]. Dental malformations are ten times more common in individuals with DS than in the general population. These include microdontia, diastema, agenesis, delayed tooth eruption, dental morphology, and a higher prevalence of bruxism [1,2,5,6]. Diastema is frequent among individuals with DS due to microdontia and agenesis. And due to a high number of existing diastema, there is a significant reduction in the prevalence of proximal carious lesions [2,3]. Theoretically, small-spaced teeth, associated with delayed tooth eruption, reduce the chance of food stagnating between the teeth and diminish the smooth surface area for colonization by cariogenic bacteria [3,9,14,15]. The same is true for bruxism, where the occlusal surfaces are often susceptible to decay and worn smooth by the grinding of teeth [1–6].

When compared with the general population, the individuals with DS are more likely to receive dental care yearly. Nevertheless, they are less likely to receive preventive and restorative care related to dental caries and more likely to have missing teeth [8,36]. The missing teeth, however, may be associated with the presence of agenesis in individuals with DS. Fung et al. [8] and Allison & Lawrence [36] did not investigate if the loss of teeth is associated with the caries disease or with the frequent agenesis in DS.

Some studies credited the low caries experience in individuals with DS to salivary composition (higher salivary pH and bicarbonate levels) and differences in the composition of microbiota (*Streptococcus mutans* counts) [10,16,19,37]. Studies reported changes in the ecosystem of the oral cavity of individuals with DS, which can result in physiological changes to the flow and composition of saliva [10,11,19,31,34].

In terms of the limitations of the selected studies, some studies presented a high risk of bias in many items [34] while others presented a risk of bias in fewer items [24,25,33] (Table 1). Risk bias was mainly due to failure to confirm diagnosis for DS through karyotype examination [11,13–15,17,20,25,32,34] and failures to report that oral examination had undergone a calibration exercise conducted through statistical tests (Cohen’s kappa coefficient) [6,11,13–15,20,31,32,34]. A proper diagnosis and an adequate calibration exercise before collecting data is important to avoid selection bias. Another limitation is that although the use of medication is quite common in people with DS, only half of the studies considered this variable in data analysis [13–15,24,31,34]. Often individuals with DS, especially children, take medicine frequently for symptoms of sinusitis, otitis, tonsillitis and other common respiratory infections among this population. In some cases, antibiotics are prescribed. These pediatric medicines contain a high level of sugar in their composition, which results in a severe cariogenic challenge in these individuals [9,20,33,37]. This is a confounder variable that may influence the prevalence or experience of caries among individuals with DS. No study has used statistical approaches for any other confounder such as socioeconomic status, age, sex, diet, brushing habits and use of fluoride. A confounder is the third factor involved in the association of the real outcome (dental caries) [38]. If it is not adjusted in epidemiological studies by statistical tests the confounder makes it difficult to determine if dental caries is due of the dependent variable (having Down Syndrome) or due the use of medication or another factor.

Another limitation of the systematic review is the inclusion of only cross-sectional studies. Cross-sectional designs lack the temporality that would be achieved by cohort designs [38]. However, cross-sectional studies should not be discarded once this design can establish the direction of the associations [38], as presented by the present meta-analysis.

The precise cause of the lower prevalence of dental caries in the population with DS is still unclear [39,40]. Although the need of treatment for dental caries is not high, these individuals must receive a dental assistance directed to the care of other needs present in this part of the population, especially periodontal disease and malocclusion, which are prevalent problems in DS.

## Conclusions

The limited scientific evidence suggests that individuals with DS have fewer dental caries than individuals without DS. This evidence can be weakened by the absence of controlling the confounders. More observational studies with larger sample sizes, proper matching between cases and controls, and better control of confounding factors such as medication, dietary habits and exposure to fluoride are needed to confirm this evidence.

## Supporting Information

S1 PRISMA Checklist PRISMA Checklist. (DOC)

S1 Table List of titles selected for full-text analysis and the reasons for exclusion. (DOC)

S1 Fig Screening of articles. Four-phase PRISMA flow diagram for study collection [23], showing the number of studies identified, screened, eligible, and included in the review and meta-analysis.
(TIFF)

S2 Fig Forest plot of meta-analysis for two cross-sectional studies evaluating the prevalence of dental caries (outcome: presence of dental caries or absence of dental caries) comparing patients with Down Syndrome (DS) and controls. Pooled effect measures [odds ratio (OR) and 95% confidence interval (CI)] indicated that patients with DS had significantly lower OR of dental caries than controls. I^2^ = 0.00. Random effect model used.
(TIFF)

S3 Fig Forest plot of meta-analysis for six cross-sectional studies evaluating the mean of DMFT in permanent dentition comparing patients with Down Syndrome (DS) and controls. Pooled effect measures [standard difference (Std diff) and 95% confidence interval (CI)] indicated that patients with DS had significantly lower mean of DMFT than controls. I^2^ = 20.32%. Random effect model used.
(TIFF)
