# Supplementary material for: Association between Dental Caries and Down Syndrome: A Systematic Review and Meta-Analysis
Source: PLoS One. 2015 Jun 18;10(6):e0127484. doi: 10.1371/journal.pone.0127484 (PMC4472226; doi:10.1371/journal.pone.0127484)
Supplement: S1 Table — (DOC) [file pone.0127484.s002.doc]

**Table 2.** List of titles selected for full-text analysis and the reasons for exclusion

| **Reference** | **Classification** |
| --- | --- |
| 1. de Castilho ARF, Marta SN (2010) Evaluation of the incidence of dental caries in patients with Down syndrome after their insertion in a preventive program. Cien Saude Colet 5:3249-3253. | Excluded: The article does not present control group. |
| 2. Cogulu D, Sabah E, Kutukculer N, Ozkinay F (2006) Evaluation of the relationship between caries indices and salivary secretory IgA, salivary pH, buffering capacity and flow rate in children with Down's syndrome. Arch Oral Biol 51:23-28. | Excluded: The article does not provide sufficient data for critical analysis. The study test association of caries with other outcomes (pH, salivary IgA, buffering capacity). |
| 3. Barnett ML, Press KP, Friedman D, Sonnenberg EM (1986) The prevalence of periodontitis and dental caries in a Downs syndrome population. J Periodontol 57:288-293. | Excluded: The article does not present control group with normal people. |
| 4. Ivancic JN, Majstorovic M, Bakarcic D, Katalinic A, Szirovicza L (2007) Dental caries in disabled children. Coll Antropol 31:321-324. | Excluded: The article not presents a control group. Data is grouped with several types of disabilities. |
| 5. Fung K, Allison PJ (2005) A comparison of caries rates in non-institutionalized individuals with and without Down syndrome. Spec Care Dentist 25:302-310. | Excluded: The article does not provide sufficient data for abstraction and critical analysis. |
| 6. Sproles AC (1973) Cyclic AMP concentration in saliva of normal children and children with Downs syndrome. J Dent Res 52:915-917. | Excluded: The outcome is properties of saliva. |
| 7. Cutress TW (1971) Dental caries in trisomy 21. Arch Oral Biol 16:1329-1344. | Excluded: The article does not provide sufficient data for critical analysis. |
| 8. Creighton WE, Wells HB (1966) Dental caries experience in institutionalized mongoloid and nonmongoloid children in North Carolina and Oregon. J Dent Res 45:66-75. | Excluded: The article does not provide sufficient data for abstraction and critical analysis. |
| 9. Hennequin M, Faulks D, Veyrune JL, Bourdiol P (1999) Significance of oral health in persons with Down syndrome: a literature review. Dev Med Child Neurol 41:275-283. | Excluded: The article is a literature review, not epidemiological observational study. |
| 10. Anders PL, Davis EL (2010) Oral health of patients with intellectual disabilities: a systematic review. Spec Care Dentist 30:110-1117. | Excluded: The article is a systematic review with several disabilities pooled together. It was not possible to extract data of individual studies for analysis only by the systematic review. |
| 11. Tesini DA (1981) An annotated review of the literature of dental caries and periodontal disease in mentally retarded individuals. Spec Care Dentist 1:75-87. | Excluded: The article is a literature review, not epidemiological observational study. |
| 12. Garg A, Utreja A, Singh SP, Angurana SK (2013) Neural tube defects and their significance in clinical dentistry: a mini review. J Investig Clin Dent 4:3-8. | Excluded: The article is a literature review, not epidemiological observational study. |
