# Supplementary figures and images for: Association between Dental Caries and Down Syndrome: A Systematic Review and Meta-Analysis
Source: PLoS One. 2015 Jun 18;10(6):e0127484. doi: 10.1371/journal.pone.0127484 (PMC4472226; doi:10.1371/journal.pone.0127484)

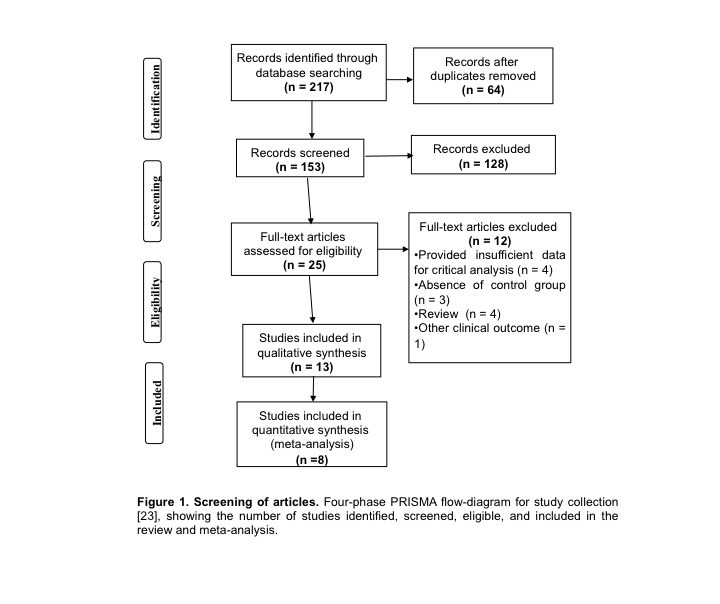

Supplement: S1 Fig — Four-phase PRISMA flow diagram for study collection [23], showing the number of studies identified, screened, eligible, and included in the review and meta-analysis. (TIFF) [file pone.0127484.s003.tiff]

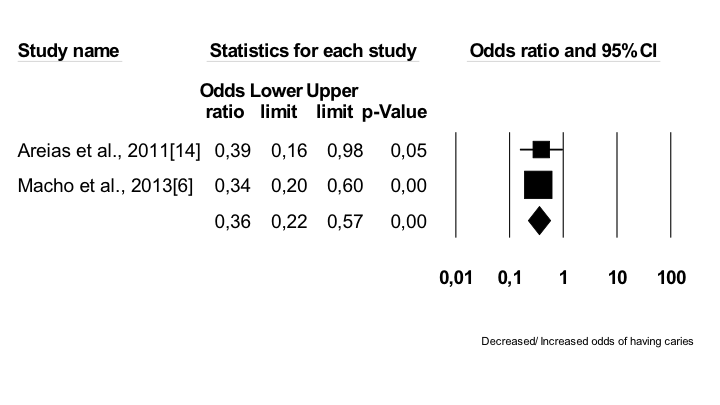

Supplement: S2 Fig — Pooled effect measures [odds ratio (OR) and 95% confidence interval (CI)] indicated that patients with DS had significantly lower OR of dental caries than controls. I2 = 0.00. Random effect model used. (TIFF) [file pone.0127484.s004.tiff]

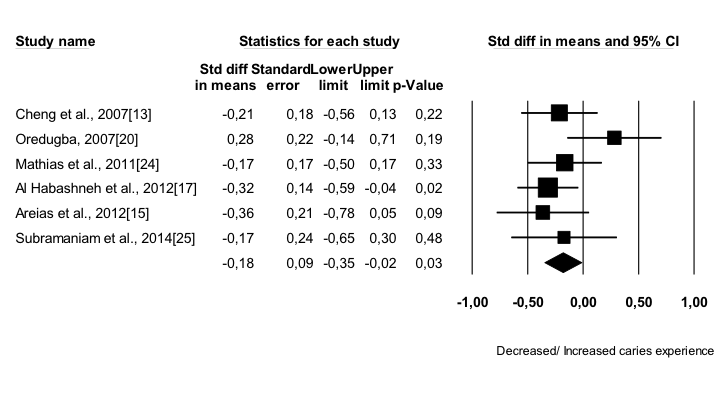

Supplement: S3 Fig — Pooled effect measures [standard difference (Std diff) and 95% confidence interval (CI)] indicated that patients with DS had significantly lower mean of DMFT than controls. I2 = 20.32%. Random effect model used. (TIFF) [file pone.0127484.s005.tiff]
